# Clinical, laboratorial and radiographic predictors of Bordetella pertussis infection☆

**DOI:** 10.1016/j.rpped.2014.06.001

**Published:** 2014-12

**Authors:** Camila Vieira Bellettini, Andressa Welter de Oliveira, Cintia Tusset, Ludmila Fiorenzano Baethgen, Sérgio Luís Amantéa, Fabrizio Motta, Aline Gasparotto, Huander Felipe Andreolla, Alessandro C. Pasqualotto

**Affiliations:** a Universidade Federal de Ciências da Saúde de Porto Alegre (UFCSPA), Porto Alegre, RS, Brazil; b Irmandade Santa Casa de Misericórdia de Porto Alegre, Porto Alegre, RS, Brazil

**Keywords:** Bordetella pertussis, Whooping cough, Infection, Coinfection

## Abstract

**OBJECTIVE:**

To identify clinical, laboratorial and radiographic predictors for *Bordetella pertussis* infection.

**METHODS:**

This was a retrospective study, which analyzed medical records of all patients submitted to a molecular dignosis (qPCR) for *B. pertussis* from September 2011 to January 2013. Clinical and laboratorial data were reviewed, including information about age, sex, signs/symptoms, length of hospitalization, blood cell counts, imaging findings, coinfection with other respiratory pathogens and clinical outcome.

**RESULTS:**

222 cases were revised. Of these, 72.5% had proven pertussis, and 60.9% were under 1 year old. In patients aging up to six months, independent predictors for *B. pertussis *infection were (OR 8.0, CI 95% 1.8-36.3; *p=*0.007) and lymphocyte count >10^4^/µL (OR 10.0, CI 95% 1.8-54.5; *p=*0.008). No independent predictors of *B. pertussis *infection could be determined for patients older than six months. Co-infection was found in 21.4% of patients, of which 72.7% were up to six months of age. Adenovirus was the most common agent (40.9%). In these patients, we were not able to identify any clinical features to detect patients presenting with a respiratory co-infection, even though longer hospital stay was observed in patients with co-infections (12 vs. 6 days; *p=*0.009).

**CONCLUSIONS:**

Cyanosis and lymphocytosis are independent predictors for pertussis in children up to 6 months old.

## Introduction

Pertussis or whooping cough is an acute respiratory tract infection caused by *Bordettella pertussis* and ranked among the 10 leading causes of childhood mortality.^1 ^An increasing number of pertussis outbreaks have been reported in the last years despite vaccination coverage. Indeed, in the last decades, the age range of affected individuals appears to have widened and the incidence of pertussis in adolescents and adults has raised.^2^^-^ 5 It is essential the prompt recognition of patients with this condition, because a delay in diagnosis could result in late onset of antibiotic treatment subsequently increasing the potential for secondary transmission.^6^ However, clinical diagnosis of whooping cough is difficult to perform, once clinical manifestations can vary according to immunization status, patient's age and the disease stages.^3^^,^ 5 ^,^ 7 ^-^ 8

Previous studies have evaluated the impact of concomitant detection of *B. pertussis* with other respiratory agents^9^^-^ 10, suggesting that *B. pertussis* infection could be more severe in this context.^11^^-^ 13 Mixed respiratory infections have been reported in children in several countries,^14^ but its actual incidence is believed to be even higher.^12^^-^ 13

The purpose of this study was to describe the clinical profile of patients with suspected *B. pertussis* infection, and to identify the clinical, laboratorial and radiographic predictors for *B. pertussis* infection. We also aimed to determine the frequency of concomitant respiratory tract infections in this population, as well as to determine if co-infections were associated with greater morbidity and/or mortality in patients with *B. pertussis* infection.

## Methods

This was a retrospective case series study performed at Santa Casa de Misericórdia de Porto Alegre, Brazil, from September 2011 to January 2013. We studied patients with suspected *B. pertussis* infection for whom a molecular diagnosis was performed at Molecular Biology Laboratory at Santa Casa. We included hospitalized patients, and patients with suspected infection that attended the emergency room or physician's office in the hospital. All patients who were tested for pertussis by real time polymerase chain reaction (qPCR) in the study period, regardless of age, were studied. Clinical samples consisted of nasopharyngeal aspirates collected by hospital's nursing team. Clinical and laboratorial data were extracted by a standardized clinical form, including information about age, sex, signs/symptoms, length of hospitalization, blood cells count, chest imaging findings, concomitant detection of other respiratory pathogens and clinical outcome. This study was approved by the local Ethic Committee (n. 115333), and all researchers signed a commitment statement to use patient's records, ensuring the confidentiality of this work.

The in house qPCR test used in this study has been available in the institution since 2011. In summary, DNA was extracted with QIAamp DNA Mini Kit (Qiagen) and stored at -80ºC. qPCR was performed using Platinum(r) SYBR(r) Green qPCR SuperMix-UDG (Invitrogen(tm)). Primers (0.2uM each) were ACGCAGTGGCCTACTACCAG and GCGGTAAGGTCGGGTAAAG. All reactions included a positive control (DNA extracted from Fiocruz ATCC strain), a negative control and an internal control (HuPO), in 25ul reactions. The qPCR conditions were 2 min at 50ºC, 5 min at 95ºC, 45 cycles of 15 sec at 95ºC and 30 sec at 60ºC, followed by 15 sec at 95ºC, 1 min at 60ºC, 30 sec at 95ºC, and 15 sec at 60ºC. A positive qPCR test was assumed as a confirmatory test for pertussis, while a negative qPCR was considered as absence of the disease. 

Statistical analyzes were performed by SPSS software (version 17.0). Patients with positive and negative qPCR results for *B. pertussis* were compared. Chi-squared test was used for categorical variables and the Mann-Whitney test was applied for continuous data. Significance was determined at α<0.05. Multivariate analysis was performed in order to identify independent predictors for *B. pertussis* infection. 

## Results

Medical records of 222 patients with suspected *B. pertussis* infection were reviewed. Among these, qPCR confirmed *B. pertussis* infection in 161 (72.5%) patients. The great majority was tested by qPCR only. Four patients were also submitted (and tested negative) to additional diagnostic test, including serology and culture. Among positive qPCR cases, 60.9% were younger than 1 year, and 52.8% were boys. Table 1 shows the age distribution according to qPCR results. The most common clinical manifestations were cough (100%), cyanosis (59.6%), plethora (49.7%), posttussive vomiting (37.9%), fever (34.2%), respiratory effort (36%) and whoop (15.5%). Most of patients with cyanosis were up to one year (81.3%), but this sign was also observed in older children and adolescents up to 13 years old. The median time interval between the beginning of symptoms and the sample submission for qPCR testing was 15 days (range, 1-60 days), versus 19 days in qPCR negative group (*p=*0.49). In the positive qPCR patients, the main alteration on chest X-ray was diffuse infiltrate (58.3%), followed by hyperinflated lungs (22.8%) and atelectasis (16.5%). Mean [range] blood cells (% where indicated) for patients with B. pertussis infection were as follows: 21,607 [6,470-99,700] (white blood cells); 11,515 [367-43,908] (lymphocytes); 53.6% [5.5-78.0%] (percentage of lymphocytes); and 503,205 [150,000-946,000] (platelets). Overall mortality in patients with *B. pertussis* infection was 2.5%; all occurring in patients 3 months of age or younger. Comparisons of the clinical and laboratorial data of patients with positive and negative qPCR results for pertussis are presented in Table 2.

**Table 1:**
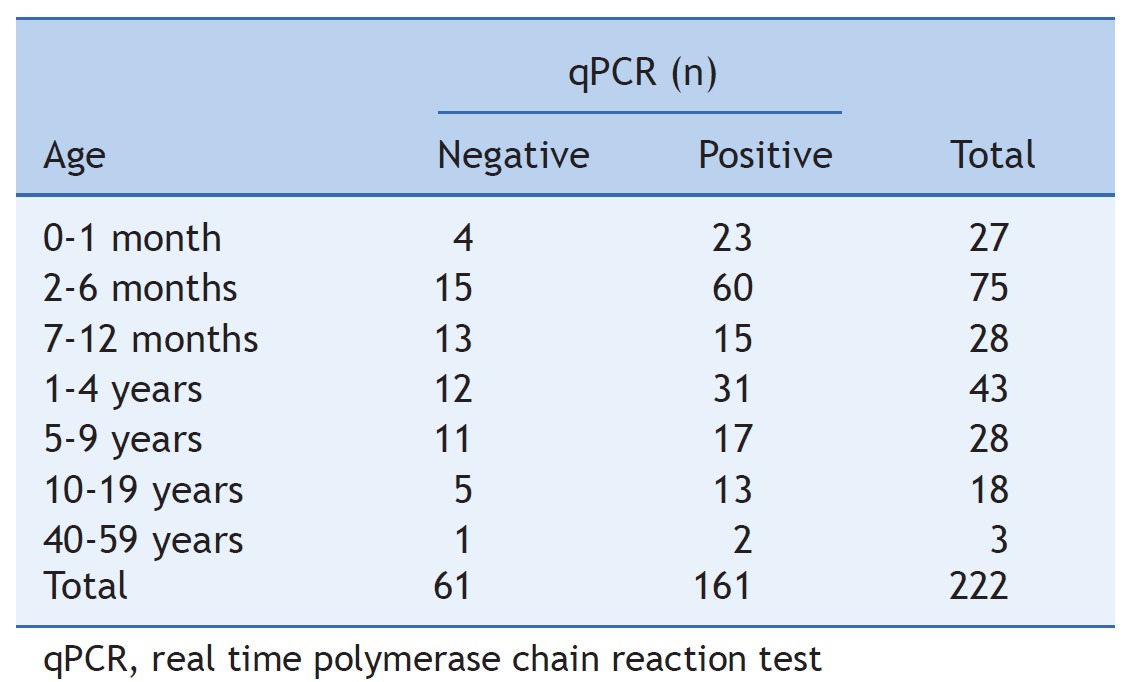
Age distribution and qPCR results in patients with suspected pertussis infection.

**Table 2:**
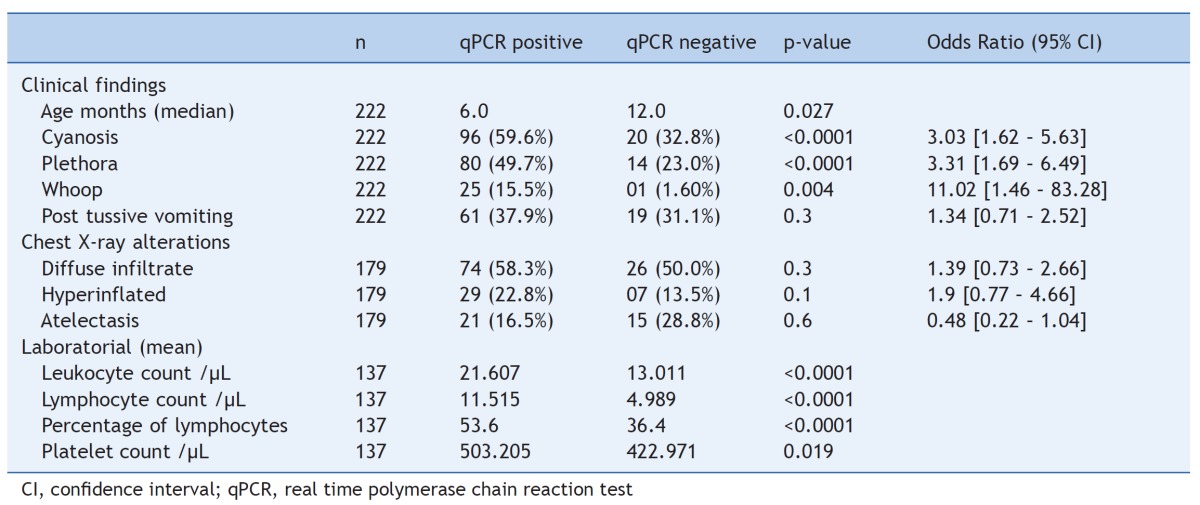
C Clinical and laboratorial data of patients with positive and negative pertussis qPCR.

From all of our patients, 164 were hospitalized (73.8%). Hospitalization rate in age up to 6 months was 94% (versus 56.6% of patients older than 6 months; *p<*0.001). Besides age, laboratorial features were also predictors of hospitalization in pertussis: lymphocytosis (*p=*0.001), leukocytosis (*p=*0.001) and thrombocytosis (*p<*0.001). There was no statistic significant difference between these groups (hospitalized vs. outpatient) in relation to qPCR result (75.1% in qPCR+ vs. 70.4% in qPCR-; *p=*0.4) and presence of co-infections (95% in coinfected vs. 91.3% in no coinfected *p=*0.5).

Immunization data was reported only in 54.5% of cases. Data available showed that 62% of positive and 31% of negative cases had received any or only one dose of the vaccine against *B. pertussis*, while only 6.5% of positive cases (versus 20.7% of negative cases) had complete immunization [in Brazil, this consists in 3 doses and 2 boosters of the vaccine]. Despite this percentage contrast, there was no statistic significant difference between the groups (*p=*0.068). Immunization received according to age group is demonstrated in Table 3.

**Table 3:**
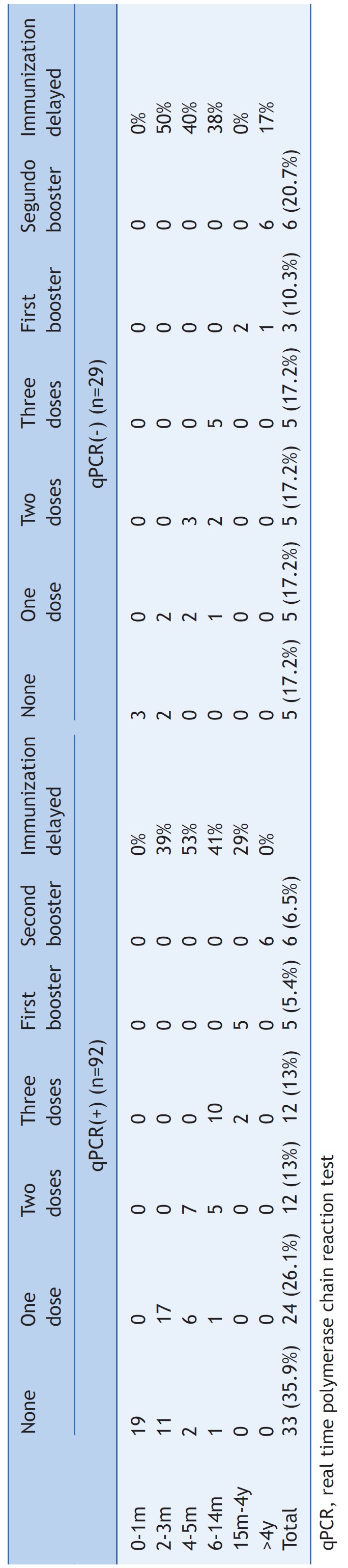
Vaccine doses received distributed by age.

We divided patients in two different groups: children aged 6 months or younger and patients older than 6 months. Predictors of *B. pertussis* infection at univariate analysis for patients aged 6 months or younger were cyanosis (OR 5.32, CI 95% 1.79-15.8; *p=*0.001), plethora (OR 4.49, CI 95% 1.54-13.1; *p=*0.004), leukocyte count (*p=*0.031), lymphocyte count (*p<*0.0001), and percentage of lymphocytes (*p=*0.002). At multivariate analysis, cyanosis (OR 8.0, CI 95% 1.8-36.3; *p=*0.007) and lymphocyte count >10^4^/µL (OR 10.0, CI 95% 1.8-54.5; *p=*0.008) were independent predictors for pertussis in children younger than 6 months of age. The only variables associated with pertussis for patients aged more than 6 months at univariate analysis were leukocyte count (*p=*0.019) and lymphocyte count (*p=*0.018). Atelectasis was associated with the presence of diagnoses other than pertussis (OR 0.2, CI 95% 0.07-0.88; *p=*0.024), in this group of patients. At multivariate analysis, no variable was associated with pertussis in patients older than 6 months of age.

**Figure 1:**
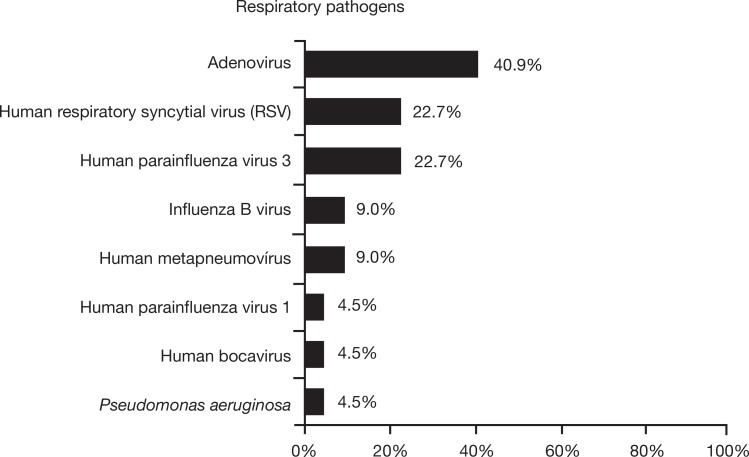
Frequency of codetection of other respiratory pathogens in patients with Bordetella pertussis infection

A total of 103 patients with confirmed *B. pertussis *infection were also tested for other respiratory pathogens [also detected by *in house* qPCR or bacterial culture]. Co-detection was found in 21.4% of these patients, 72.7% of whom were 6 months of age or younger, 13.6% were older than 6 months but younger than 1 year, and 13.6% were between 1 and 4 years old. Fig. 1 displays the frequency of co-detection of other respiratory pathogens in patients with pertussis. There was no statistically significant difference between age and pathogens distribution (*p=*0.71). Patients with co-detection of pertussis and other respiratory pathogens had prolonged stay in the hospital (12 vs. 6 days; *p=*0.009) and more atelectasis on chest X-ray (38.1% vs. 16.7%; OR 3.3, CI 95% 1.1-9.5; *p=*0.023). 

## Discussion

In this work, we reviewed the clinical charts of 222 patients suspected of *B. pertussis* infection. Of these, the diagnosis of pertussis was confirmed in 72.5% by the means of qPCR. Considering that only four patients were also submitted to another diagnostic test for comparison (serology or culture), we assumed that negative qPCR indicated absence of the disease. Even though, all patients were in a similar moment of disease by the time of molecular diagnosis. 

As expected, most of these children were aged 1 year of age or less (60.9%), which is in accordance with previous data that reported rates between 50% and 64.6% in this age group.^15^^-^ 16 Recently, it was reported a significant increase in the incidence of pertussis in adolescents and adults.^2^^-^ 3 However, in this study, few individuals with positive qPCR were older than 15 years (1.8%). This fact could be justified by the heterogeneity in the clinical manifestations of pertussis, in association with a low index of suspicion among clinicians for such condition in adults.^3^^,^ 17 ^-^ 20

It is well known that pertussis may present different manifestations according to patients' age, among children.^3^^,^ 5 ^,^ 8 ^,^ 21 ^-^ 22 In this work, we stratified patients in two separated groups: children aged 6 months or less and patients older than 6 months. Our results suggest that classical manifestations of pertussis vary according to patients' age and presumably to the number of vaccine doses against *B. pertussis* they receive. For instance, in younger children (i.e., <6 months of age), pertussis was associated with the presence of cyanosis, plethora, leukocyte count, lymphocyte count and lymphocytes percentage. Multivariate analysis showed that cyanosis and lymphocyte count were able to predict pertussis in this patient group. On the other hand, older patients (i.e., >6 months of age) presented with less classical symptoms of pertussis, and no variable could be independently associated with pertussis. This is similar to previously reported data, showing that patients who were immunized against pertussis may develop the disease with atypical presentations.^5^^,^ 6 ^,^ 18 ^-^ 20 The most common typical finding, which appears to be present in all age groups, is prolonged cough.^7^^,^ 18 ^-^ 19

Cyanosis is already known as a pertussis classic symptom,^2^^,^ 8 and leukocytosis attributable to lymphocytosis is also recognized as a hallmark of pertussis.^7^^,^ 23 ^-^ 25 In this study, lymphocytosis was not only a hallmark, but also was an independent predictor for *B. pertussis* infection in young infants. The cutoff point (10^4^/µL) was similar with those found in previous studies.^24^^,^ 25 These data suggest that the occurrence of cyanosis should increase the pediatrician´s suspicion of *B. pertussis* infection, particularly in the presence of lymphocytosis in a young child.

Co-detection of *B. pertussis* with other respiratory agents has already been reported,^12^^,^ 22 ^,^ 26 ^-^ 29 and it may be actually underestimated.^12^^-^ 13 It was reported that pertussis toxin may suppress innate immune response and sensitize the host to a secondary respiratory pathogen.^13^ In our study, we found an incidence of 21.4% of mixed infections; the most frequent pathogens associated with *B. pertussis* were adenovirus, RSV and parainfluenza type 3 virus. These viruses (especially RSV) were also prevalent in some previous reports.^22^^,^ 26 ^-^ 29 The evaluation of laboratorial data of infants up to 6 months showed that lymphocyte percentage is significant different (and higher) among patients with respiratory pathogens co-detection and patients with only pertussis identification. Previous studies related mixed infection with higher severity of the disease.^11^^-^ 13 In this work, patients with co-detection pathogens had a longer period of hospitalization, reinforcing the relationship between mixed infection and a more severe disease. On the other hand, as reported in other works, we did not reliably distinguish clinical features of infants with mixed infection from those with only pertussis detection.^11^^-^ 12 ^,^ 22 ^,^ 26 ^-^ 27 Furthermore, it is important to note that less than 50% of our patients were tested to other patogens, and these results must be seen with caution.

This study has some limitations that should be mentioned. In view of its retrospective design and the samples were obtained by convenience, some data were missing at analysis, particularly those related to immunization history, clinical and laboratorial exams, and co-infection tests. Despite that were to study a large number of patients, and for most of the other variables data missing was minimal. Another limitation is related to the limited number of adult patients included in the study - therefore, our conclusion cannot be extrapolated to this patient group. 

An increasing number of pertussis outbreaks have been reported despite vaccination coverage.^2^^-^ 5 It is unclear the factor that contribute to pertussis resurgence, but waning immunity, improved surveillence and diagnosis, as well as adaptation of circulating Bordetella pertussis strains could be involved.^5^ Even though, the most important and effective way to control pertussis remains vaccination.^5^ Ensuring wider immunization coverage, as well as developing newer strategies to avoid waning immunity after pertussis vaccination and disease - as booster vaccines in adolescents and adults - is something highly desirable.^2^^,^ 4 ^,^ 5

*B. pertussis* infection is a common disease affecting all ages. Clinicians should be aware of this condition and perform a prompt diagnosis and initiate an early treatment, avoiding secondary transmission of the disease. This work showed that young children may manifest clinical and laboratorial features that may help the clinician to suspect the presence of pertussis. Although children with 6 months or more and adults may present with atypical manifestations of pertussis, the diagnosis must be always considered in these patients, in the presence of prolonged cough.
