# Supplementary material for: Clinical, laboratorial and radiographic predictors of Bordetella pertussis infection
Source: Rev Paul Pediatr. 2014 Dec;32(4):292–8. doi: 10.1016/j.rpped.2014.06.001 (PMC4311781; doi:10.1016/j.rpped.2014.06.001)
Supplement: Translation

# Translation: Preditores clínicos, laboratoriais e radiográficos para infecção por Bordetella pertussis☆

**Authors:** Camila Vieira Bellettini, Andressa Welter de Oliveira, Cintia Tusset, Ludmila Fiorenzano Baethgen, Sérgio Luís Amantéa, Fabrizio Motta, Aline Gasparotto, Huander Felipe Andreolla, Alessandro C. Pasqualotto

**Affiliations:** a Universidade Federal de Ciências da Saúde de Porto Alegre (UFCSPA), Porto Alegre, RS, Brasil; b Irmandade Santa Casa de Misericórdia de Porto Alegre, Porto Alegre, RS, Brasil

**Keywords:** *Bordetella pertussi*s, Coqueluche, Infecção, Coinfecção

## Abstract

**OBJETIVO:**

Identificar preditores clínicos, laboratoriais e radiológicos da infecção por *Bordetella pertussis*.

**MÉTODOS:**

Trabalho retrospectivo, com análise de prontuários clínicos de todos os indivíduos submetidos ao diagnóstico molecular (qPCR) para *B. pertussis* de setembro de 2011 à janeiro de 2013. Foram revistos dados clínicos e laboratoriais, incluindo informações sobre idade, sexo, sinais/sintomas, tempo de hospitalização, contagens de células sanguíneas, exames de imagem, co-infecção com outros patógenos respiratórios, e evolução clínica.

**RESULTADOS:**

222 casos foram revistos, do quais 72,5% tinham coqueluche confirmada, sendo 60,9% menores de um ano de idade. Foram observados preditores independentes para *B. pertussis* em pacientes com menos de seis meses de idade. Nesses casos, os preditores identificados foram cianose (OR 8,0; CI 95% 1,8-36,3; *p=*0,007) e contagem de linfócitos >10^4^/µL (OR 10,0, CI 95% 1,8-54,5; *p=*0,008). Preditores de coqueluche não puderam ser determinados para crianças maiores de 6 meses de idade. Coinfecção foi encontrada em 21,4% dos pacientes, dos quais 72,7% tinham até seis meses de idade, sendo que o adenovírus foi o agente mais comum (40,9%). Nesses indivíduos, não foram observadas características clíncias capazes de distinguir pacientes com co-infecção, porém foi verificado um maior tempo de internação hospitalar nos pacientes com mais de um agente infeccioso detectado (12 vs. 6 dias; *p=*0,009).

**CONCLUSÕES:**

Cianose e linfocitose são preditores independentes para coqueluche em crianças com até seis meses de idade.

## Introdução

Pertussis ou coqueluche é uma infecção aguda do trato respiratório causada por *Bordetella pertussis*, e classificada entre as 10 principais causas de mortalidade infantil.^1^ Um número crescente de surtos de coqueluche foi relatado nos últimos anos, apesar da cobertura vacinal. De fato, nas últimas décadas, a faixa etária dos indivíduos afetados parece ter sido ampliada, e a incidência de coqueluche em adolescentes e adultos tem aumentado.^2^^-^ 5 É essencial o reconhecimento imediato dos pacientes com essa condição, pois a demora no diagnóstico pode resultar em início tardio do tratamento antibiótico, posteriormente aumentando o potencial de transmissão secundária.^6^ No entanto, o diagnóstico clínico de coqueluche é de difícil execução, já que as manifestações clínicas podem variar de acordo com o estado de imunização, idade do paciente e as fases da doença.^3^^,^ 5 ^,^ 7 ^-^ 8

Estudos anteriores já haviam avaliado o impacto da detecção concomitante de *B. pertussis* com outros agentes respiratórios,^9^^-^ 10 sugerindo que a infecção por *B. pertussis* pode ser mais grave nesse contexto.^11^^-^ 13 Infecções respiratórias mistas foram relatadas em crianças em vários países,^14^ mas acredita-se que sua verdadeira incidência seja ainda maior.^12^^-^ 13

O objetivo desse estudo foi descrever o perfil clínico de pacientes com suspeita de infecção por *B. pertussis*, e identificar os preditores clínicos, laboratoriais e radiográficos para a infecção por *B. pertussis*. Também tivemos como objetivo determinar a frequência de infecções concomitantes do trato respiratório nessa população, bem como determinar se coinfecções estavam associadas a maior morbidade e/ou mortalidade em pacientes com infecção por *B. pertussis*.

## Métodos

Estudo retrospectivo de série de casos realizado na Santa Casa de Misericórdia de Porto Alegre, Brasil, de setembro de 2011 a janeiro de 2013. Estudamos pacientes com suspeita de infecção por *B. pertussis* submetidos a diagnóstico molecular realizado no Laboratório de Biologia Molecular da Santa Casa. Foram incluídos pacientes internados e pacientes com suspeita de infecção que buscaram atendimento no pronto-socorro ou em consultório médico no hospital.

Todos os pacientes que foram testados para coqueluche por reação da polimerase em cadeia em tempo real (PCRq) durante o período de estudo, independente da idade, foram estudados. Analisamos material de secreção nasofaríngea coletados pela equipe de enfermagem do hospital. Os dados clínicos e laboratoriais foram extraídos em um formulário clínico padronizado, incluindo informações sobre idade, sexo, sinais/sintomas, tempo de internação, hemograma, exames de imagem do tórax, detecção concomitante de outros patógenos respiratórios e evolução clínica. Esse estudo foi aprovado pelo Comitê de Ética da instituição (n^o^. 115333), e todos os pesquisadores assinaram um termo de compromisso para utilizar o prontuário do paciente, garantindo a confidencialidade desse estudo. 

O teste de PCRq in-house utilizado nesse estudo está disponível na instituição desde 2011. Em resumo, o DNA foi extraído com *QIAamp DNA Mini Kit* (Qiagen, Califórnia, Estados Unidos) e armazenado a -80°C. qPCR foi realizada utilizando *Platinum(r) SYBR(r) Green qPCR SuperMix-UDG* (Invitrogen(tm), Life Technologies, Nova York, Estados Unidos). *Primers* (0.2 uM cada) foram ACGCAGTGGCCTACTACCAG e GCGGTAAGGTCGGGTAAAG. Todas as reações incluíam um controle positivo (DNA extraído de cepa ATCC gentilmente cedida pela Fiocruz), controle negativo e um controle interno (HuPO), em reações de 25 ul. As condições da qPCR foram 2 minutos a 50°C, 5 minutos a 95°C, 45 ciclos de 15 segundos a 95°C e 30 segundos a 60°C, seguidos de 15 segundos a 95°C, 1 minuto a 60°C, 30 segundos a 95°C e 15 segundos a 60°C. Um teste positivo de PCRq foi considerado um teste confirmatório para a coqueluche, enquanto um PCRq negativo foi considerado como a ausência da doença. 

As análises estatísticas foram realizadas utilizando-se o software SPSS, versão 17 (*SPSS Statistics for Windows*, Chicago, Estados Unidos). Os pacientes com resultados positivos e negativos de PCRq para *B. pertussis* foram comparados. O teste do qui-quadrado foi utilizado para variáveis ​​categóricas, e o teste de Mann-Whitney foi utilizado para dados contínuos. A significância foi estabelecida em α<0,05. A análise multivariada foi realizada para identificar preditores independentes para a infecção por *B. pertussis*. 

## Resultados

Os registros médicos de 222 pacientes com suspeita de infecção por *B. pertussis *foram revisados. Entre esses, PCRq confirmou infecção por *B. pertussis* em 161 (72,5%) pacientes. A maioria foi testada apenas por PCRq. Quatro pacientes foram submetidos (com resultado negativo) a teste diagnóstico adicional, incluindo sorologia e cultura. Entre os casos positivos de PCRq, 60,9% tinham menos de 1 ano de idade, e 52,8% eram do sexo masculino. A tabela 1 mostra a distribuição etária de acordo com resultados de PCRq. As manifestações clínicas mais comuns foram tosse (100%), cianose (59,6%), pletora (49,7%), vômitos pós-tosse (37,9%), febre (34,2%), esforço respiratório (36%) e tosse convulsa (15,5%). 

**Tabela 1:** Distribuição etária e resultados da qPCR em pacientes com suspeita de infecção por pertussis

A maioria dos pacientes com cianose tinha até um ano de idade (81,3%), mas esse sintoma também foi observado em crianças mais velhas e adolescentes de até 13 anos. O tempo de intervalo mediano entre o início dos sintomas e o envio das amostras para testes de PCRq foi de 15 dias (variação de 1-60 dias), contra 19 dias no grupo PCRq negativo (*p=*0,49). Nos pacientes PCRq positivos, a principal alteração na radiografia de tórax foi infiltrado difuso (58,3%), seguido por pulmões hiperaerados (22,8%) e atelectasia (16,5%). As médias [variação] de células sanguíneas (% quando indicadas) foram as seguintes: 21.607 [6.470-99.700] (células brancas do sangue); [11.515] 367-43.908 (linfócitos); 53,6% [5,5-78,0%] (porcentagem de linfócitos); e 503.205 [150,000-946,000] (plaquetas).

A mortalidade geral em pacientes com infecção por *B. pertussis* foi de 2,5%; todos tinham 3 meses de idade ou menos. A comparação dos dados clínicos e laboratoriais de pacientes com resultados positivos e negativos de PCRq para coqueluche é apresentada na tabela 2.

**Tabela 2:** Dados clínicos e laboratoriais de pacientes com resultados positivo e negativo de qPCR para pertussis

Do total de pacientes, 164 foram hospitalizados (73,8%). A taxa de hospitalização em idade até 6 meses foi de 94% (contra 56,6% em pacientes com mais de 6 meses; *p*<0,001). Além da idade, características laboratoriais também foram preditoras de hospitalização em coqueluche: linfocitose (*p*=0,001), leucocitose (*p*=0,001) e trombocitose (*p*<0,001). Não houve diferença estatisticamente significante entre os grupos (hospitalizado vs. ambulatorial) em relação ao resultado da PCRq (75,1% em PCRq+ *vs*. 70,4% em PCRq-, *p*=0,4) e presença de coinfecções (95% em coinfectados *vs*. 91,3% em não coinfectados, *p*=0,5).

Dados de imunização foram relatados em apenas 54,5% dos casos. Os dados disponíveis mostram que 62% dos casos positivos e 31% dos negativos receberam nenhuma ou apenas uma dose da vacina contra a *B. pertussis*, enquanto apenas 6,5% dos casos positivos (contra 20,7% dos casos negativos) tiveram a imunização completa no Brasil, isso consiste em 3 doses e 2 reforços da vacina. Apesar desse contraste de porcentagens, não houve diferença estatisticamente significativa entre os grupos (*p=*0,068). A imunização recebida de acordo com a faixa etária é mostrada na tabela 3. 

**Tabela 3:** Doses de vacina recebidas distribuídas por idade

Os pacientes foram divididos em dois grupos: crianças de até 6 meses, e pacientes com mais de 6 meses. Preditores de infecção por *B. pertussis* na análise univariada para pacientes com idade até 6 meses foram cianose (OR: 5,32, IC 95%: 1,79-15,8, *p=*0,001), pletora (OR: 4,49, IC 95%: 1,54-13,1, *p*=0,004), contagem de leucócitos (*p=*0,031), contagem de linfócitos (*p*<0,0001), e a porcentagem de linfócitos (*p=*0,002). 

Na análise multivariada, cianose (OR: 8,0, IC 95%: 1,8-36,3, *p=*0,007) e contagem de linfócitos >10^4^/mL (OR: 10,0, IC 95%: 1,8-54,5, *p=*0,008) foram preditores independentes para coqueluche em crianças menores de 6 meses de idade. As únicas variáveis ​​associadas à coqueluche em pacientes com idade superior a 6 meses na análise univariada foram contagem de leucócitos (*p*=0,019) e contagem de linfócitos (*p=*0,018). A presença de atelectasia foi associada à presença de outros diagnósticos diferentes de coqueluche (OR: 0,2, IC 95%: 0,07-0,88, *p=*0,024) nesse grupo de pacientes. Na análise multivariada, nenhuma variável estava associada à coqueluche em pacientes com mais de 6 meses de idade.

Um total de 103 pacientes com infecção por *B. pertussis* confirmada também foram testados para outros patógenos respiratórios. Co-detecção foi encontrada em 21,4% desses pacientes, dos quais 72,7% tinham 6 meses de idade ou menos, 13,6% tinham idade superior a 6 meses, mas menos de 1 ano, e 13,6% tinham entre 1 e 4 anos de idade. A figura 1 mostra a frequência de codetecção de outros patógenos respiratórios em pacientes com coqueluche. Não houve diferença estatisticamente significativa entre a idade e a distribuição de patógenos (*p*=0,71). Pacientes com co-detecção de coqueluche e outros patógenos respiratórios apresentaram maior tempo de internação hospitalar (12 *vs*. 6 dias; *p*=0,009) e mais atelectasia na radiografia de tórax (38,1% *vs*. 16,7%; OR: 3,3, IC 95%: 1,1-9,5, *p*=0,023).

**Figura 1:** Frequência de codetecção de outros patógenos respiratórios em pacientes com infecção por Bordetella pertussis.

## Discussão

Nesse trabalho, analisamos os prontuários de 222 pacientes com suspeita de infecção por *B. pertussis*. Desses, o diagnóstico de coqueluche foi confirmado em 72,5% por meio de PCRq. Considerando que apenas quatro pacientes também foram submetidos a outro teste diagnóstico para comparação (sorologia ou cultura), concluímos que um resultado negativo da PCRq indica ausência da doença. Entretanto, todos os pacientes estavam em um momento semelhante da doença quando foi realizado o diagnóstico molecular.

Como esperado, a maioria dessas crianças tinha um ano de idade ou menos (60,9%), o que está de acordo com dados anteriores que relataram taxas entre 50% e 64,6% nessa faixa etária.^15^^-^ 16 Recentemente, foi relatado um aumento significativo na incidência de coqueluche em adolescentes e adultos.2-3 Entretanto, nesse estudo, poucos indivíduos com PCRq positivo tinham mais de 15 anos de idade (1,8%). Esse fato pode ser justificado pela heterogeneidade nas manifestações clínicas da coqueluche, em associação a um baixo índice de suspeita entre os médicos para tal condição em pacientes adultos.^3^^,^ 17 ^-^ 20

É um fato bem conhecido que a coqueluche pode apresentar diferentes manifestações de acordo com a idade do paciente, quando na faixa etária pediátrica.^3^^,^ 5 ^,^ 8 ^,^ 21 ^-^ 22 Nesse trabalho, estratificamos os pacientes em dois grupos distintos: crianças de 6 meses de idade ou menos, e pacientes com mais de 6 meses. Nossos resultados sugerem que as manifestações clássicas da coqueluche variam de acordo com a idade dos pacientes e, presumivelmente, de acordo com o número de doses de vacina contra *B. pertussis* que receberam. Por exemplo, em crianças mais jovens (ou seja, ≤6 meses de idade), coqueluche estava associada à presença de cianose, pletora, contagem de leucócitos, contagem de linfócitos e porcentagem de linfócitos. A análise multivariada mostrou que cianose e a contagem de linfócitos eram capazes de prever a presença de coqueluche nesse grupo de pacientes. 

Por outro lado, pacientes mais velhos (ou seja, idade >6 meses) apresentaram menos sintomas clássicos da coqueluche, e nenhuma variável pode ser associada de forma independente à coqueluche. Esses dados são semelhantes aos dados relatados anteriormente, mostrando que os pacientes que foram imunizados contra a coqueluche podem desenvolver a doença com apresentações atípicas.^5^^,^ 6 ^,^ 18 ^-^ 20 O achado típico mais comum, que parece estar presente em todas as faixas etárias, é a tosse prolongada.^7^^,^ 18 ^-^ 19

Sabe-se que a cianose é um dos sintomas clássicos da coqueluche,^2^^,^ 8 e a leucocitose atribuível à linfocitose também é reconhecida como uma característica de coqueluche.^7^^,^ 23 ^-^ 25 Nesse estudo, a linfocitose não foi apenas uma característica, mas também um preditor independente para a infecção por *B. pertussis *em lactentes jovens. O ponto de corte (10^4^/μL) foi semelhante aos encontrados em estudos anteriores.^24^^,^ 25 Esses dados sugerem que a ocorrência de cianose deve aumentar a suspeita do pediatra de infecção por *B. pertussis*, particularmente na presença de linfocitose em uma criança pequena.

A co-detecção de *B. pertussis* com outros agentes respiratórios já foi relatada anteriormente,^12^^,^ 22 ^,^ 26 ^-^ 29 e pode ser na verdade subestimada.^12^^-^ 13 Foi relatado que a toxina da coqueluche pode suprimir a resposta imune inata e sensibilizar o hospedeiro a um patógeno respiratório secundário.^13^ Em nosso estudo, encontramos uma incidência de 21,4% de infecções mistas; os patógenos mais frequentes associados a *B. pertussis* foram adenovírus, VRS, e vírus da Parainfluenza tipo 3. Esses vírus (especialmente VRS) também foram encontrados em alguns estudos anteriores.^22^^,^ 26 ^-^ 29

A avaliação de dados laboratoriais de lactentes de até 6 meses de idade mostrou que a porcentagem de linfócitos é significativamente diferente (e maior) entre pacientes com co-detecção de outros patógenos respiratórios e pacientes com identificação apenas de coqueluche. Estudos anteriores associaram a presença de infecção mista à maior gravidade da doença.^11^^-^ 13 Nesse trabalho, os pacientes com co-detecção de patógenos apresentaram um maior período de internação hospitalar, reforçando a associação entre a infecção mista e doença mais grave. Por outro lado, conforme relatado em outros trabalhos, não pudemos distinguir de forma confiável as características clínicas de crianças com infecção mista quando comparadas àquelas com detecção apenas de coqueluche.^11^^,^ 12 ^,^ 22 ^,^ 26 ^-^ 27 Além disso, é importante notar que menos de 50% de nossos pacientes foram testados para outros patógenos, e esses resultados devem ser considerados com cautela.

Esse estudo apresenta algumas limitações que devem ser mencionadas. Tendo em vista seu caráter retrospectivo e o fato de que as amostras foram obtidas por conveniência, alguns dados estavam faltando na análise, particularmente aqueles relacionados ao histórico de imunizações, exames clínicos e laboratoriais, e os testes de coinfecção. Apesar disso, conseguimos estudar um grande número de pacientes e, na maior parte das outras variáveis, a informação ausente era mínima. Outra limitação foi o pequeno número de pacientes adultos incluídos no estudo - logo, nossas conclusões não podem ser extrapoladas para este grupo de pacientes.

Um número crescente de surtos de coqueluche foi relatado, apesar da cobertura vacinal.^2^^-^ 5 Não está claro qual é o fator contribuinte para o ressurgimento da coqueluche, mas o enfraquecimento da imunidade, melhoria do rastreamento e do diagnóstico, bem como a adaptação de cepas circulantes de *Bordetella pertussis* podem estar ser envolvidos.^5^ Entretanto, a forma mais importante e eficaz para controlar a coqueluche continua sendo a vacinação.^5^ Garantir a cobertura vacinal mais ampla, bem como o desenvolvimento de estratégias mais recentes para evitar o declínio da imunidade após a vacinação contra coqueluche e a doença - como doses de reforço em adolescentes e adultos - é algo altamente desejável.^2^^,^ 4 ^,^ 5

A infecção por* B. pertussis *é uma doença comum que afeta todas as faixas etárias. Os médicos devem estar cientes dessa condição, realizar o diagnóstico em tempo hábil e iniciar o tratamento precocemente, evitando a transmissão secundária da doença. Esse trabalho mostrou que as crianças podem manifestar características clínicas e laboratoriais que podem auxiliar o médico na suspeita da presença de coqueluche. Embora as crianças com 6 meses de idade ou mais e adultos possam apresentar manifestações atípicas da coqueluche, o diagnóstico sempre deve ser considerado nesses pacientes, na presença de tosse prolongada.
